# Pulmonary Tuberculosis in a Young Pregnant Female: Challenges in Diagnosis and Management

**DOI:** 10.1155/2008/628985

**Published:** 2008-03-18

**Authors:** Manogna Maddineni, Mukta Panda

**Affiliations:** Department of Medicine, University of Tennessee College of Medicine Chattanooga, Chattanooga, TN 37403, USA

## Abstract

*Background*. With the world becoming a global village, tuberculosis is no longer limited to endemic areas. Our case emphasizes the impact of immigration on infectious disease epidemiology and challenges associated with diagnosis and treatment in pregnancy. *Case*. A 21-year-old Hispanic female presented in preterm labor and was found to be hypoxic. Chest X-ray revealed a paratracheal mass which a CT scan confirmed. PPD test was positive. Bronchoalveolar lavage did not reveal acid-fast bacilli and biopsy revealed caseating granulomas. Diagnosis and treatment were challenging due to constraints in radiological investigations, lack of initial evidence of acid-fast bacilli, and toxic profile of medications. Due to her high risk, she was started on antituberculosis regimen. The diagnosis was confirmed on Day 26 when *Mycobacterium tuberculosis* was isolated by DNA probe. *Conclusion*. A high index of suspicion is required to recognize the changing face and disease spectrum of tuberculosis and initiate treatment for better outcomes.

## 1. INTRODUCTION

The phenomenon of immigration has an immense impact on the health care of population in the United States. Cases of tuberculosis (TB) among foreign-born nationals, currently living in the United States, account for more than half of the total. Recognition of this fact helps in early detection. Atypical presentation may lead to misdiagnosis or a delay in diagnosis. Also the absence of systemic symptoms does not rule out TB. Radiological imaging, sputum smear, and PPD only aid in the diagnosis but a high degree of suspicion is required to ascertain an accurate diagnosis. Establishing an early diagnosis of TB infection and disease in a pregnant woman is important as it affects the health of both mother and infant [1].

## 2. CASE REPORT

A 21-year-old Hispanic female with a 32-week twin pregnancy presented in preterm labor. She had immigrated to the United States two years prior, and her pregnancy course was uncomplicated except for complaints of an occasional nonproductive cough. She was a nonsmoker, denied any history of TB exposure, and was HBsAg and HIV negative. On general exam, she was tachypneic and anxious but in no apparent respiratory distress. She was found to have a low oxygen saturation of 89% on room air with a respiratory rate of 30/min and her blood pressure was 127/76 mmHg. Lung exam was clear to auscultation. Her uterine fundal height corresponded to 32-week twin pregnancy and she had bilateral pedal edema. She was also noted to have uterine contractions every 1–3 minutes.

**Figure 1:**
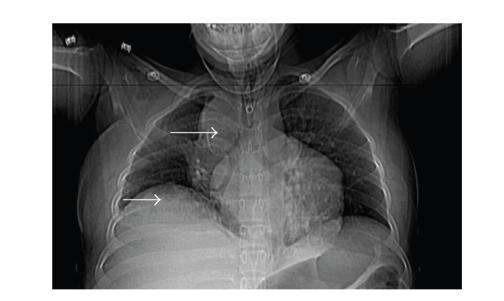
Chest X-ray. Right paratracheal soft tissue opacity measuring approximately 4 × 4 cm. Elevation of the right hemidiaphragm with a linear density in the right lower lung consistent with atelectasis.

She was initially admitted to the labor and delivery unit in our hospital and subsequently transferred to the medical ICU. ABG showed pH of 7.41, PaCO_2_ of 29, PaO_2_ of 64, bicarbonate of 18, and the base excess of −6, saturating at 93% on 21% FiO_2_. CBC showed WBC 9.9 th/mm^3^, Hb, and Hct 10.8 g/dl and 32.8%, respectively, Platelets 432 th/mm^3^. The chest X-ray demonstrated a right paratracheal soft tissue opacity measuring approximately 4 × 4 cm with a slight elevation of the right hemidiaphragm with a linear density in the right lower lung consistent with atelectasis (see Figure 1). No prior X-ray was available for comparison. Patient was put in respiratory isolation early on and the pulmonary team was consulted. CT showed a large extensive mediastinal soft tissue mass in the right paratracheal region extending into the hilar and subcarinal region. An associated atelectasis on the right upper and lower lobe with questionable alveolar infiltrate in the right lower lobe was also found. An abdominal ultrasound showed twin-twin transfusion with reversed end-diastolic flow on umbilical artery doppler in twin B and the patient had an emergent low transverse cesarean section the next day with delivery of two male infants. Infant A had Apgars of 7 and 9, and infant B had Apgars of 0, 4, and 5, respectively. Both infants went to the NICU, and the patient returned to the MICU.

A PPD was placed which was positive at 10 mm after 72 hours. Successful samples of sputum could not be obtained but a bronchoscopy with endobronchial biopsy and bronchoalveolar lavage (BAL) was done. Bronchoscopy showed a trachea with diffuse mucosal swelling and the mucosal abnormalities persisted throughout the right bronchial tree. She also had irregular tumor growth from the anterior wall of the proximal bronchus intermedius and the base of the proximal right upper lobe bronchus. These two growths were sampled and the infectious disease team was consulted. There was an abundant growth of *Staphylococcus aureus* but no acid-fast bacilli (AFB), fungus, or hyphae from the BAL. The patient was started on Vancomycin and Clindamycin to cover Methicillin-resistant *Staphylococcus aureus* (MRSA) which was later changed to Moxifloxacin based on sensitivities. Transbronchial biopsy of the right bronchus intermedius showed inflammatory and squamous debris with ulceration, reactive squamous epithelium, and colonizing coccal bacteria. Endobronchial biopsy of the proximal bronchus wall showed caseating granulomatous inflammation.

The treatment of this young Hispanic immigrant female with an occasional cough, positive PPD, questionable alveolar infiltrate, caseating granulomatous inflammation and no significant evidence of TB infection posed a definite challenge. Given her high risk for TB, she was empirically started on INH, Rifampin, Ethambutol, and Pyrazinamide. It was only on Day 26 that *Mycobacterium tuberculosis* was isolated by DNA probe from her right lung tissue which was subsequently confirmed on culture. The infants were started on INH prophylaxis after placental pathology did not demonstrate evidence of TB. Patient was discharged to home in a stable condition to be followed up at the local TB center for direct observation therapy.

## 3. DISCUSSION

Among the communicable diseases, TB is the second leading cause of death worldwide, killing nearly 2 million people each year [2]. The World Health Organization estimates that 2 billion people have latent TB, in addition to the 3 million people worldwide who die each year due to TB. After a resurgence of TB in the United States between 1985–1992, the decline noticed during 1993–2005 has slowed down causing concerns that the progress achieved in eliminating TB is slowing down [3]. Immigration demographics demonstrate that Asians, blacks, and Hispanics essentially bear the burden of TB in immigrants [3]. The number of TB cases due to foreign-born individuals has increased each year since 1993. In 1996, 10% of the 20,973 US TB cases were among foreign-born Hispanic persons, with the four states bordering Mexico accounting for 83% of foreign-born Hispanic cases. Mexicans accounted for 22.9% of foreign-born patients or 8.4% of all US cases and persons born in Central America comprised an additional 4.7% of foreign-born patients or 1.7% of all US cases. Guatemala and El Salvador contributed to nearly 75% of these cases and the remaining were from the rest of Central America [4]. In 2005, when compared between foreign-born persons in the United States and US-born persons, the TB rates were 8.7 times higher in immigrants; with Hispanics, Blacks, and Asians having TB rates 7.3, 8.3, and 19.6 times higher, respectively, than those of whites [3].

*M. tuberculosis* is transmitted by airborne droplet nuclei, which may contain fewer than 10 bacilli and humans are the only known reservoir for *M. tuberculosis* [5]. Individuals at high risk for *M. tuberculosis* infection in industrialized countries include (i) individuals likely to be exposed to or become infected with *M. tuberculosis*: close contacts of a patient with infectious TB, foreign-born individuals from high-incidence areas, the elderly, residents of long-term care facilities (e.g., correctional facilities and nursing homes), IV drugs abusers, other groups identified locally as having increased prevalence of TB (e.g., migrant farm workers or homeless persons), and individuals who may have occupational exposure to TB; and (ii) individuals at high risk of developing TB disease once infected: individuals recently infected with *M. tuberculosis* (within the past 2 years), HIV-infected individuals, individuals with immunosuppressing conditions or medication use, individuals with a history of inadequately treated TB, and infants. Chances that an individual acquires infection depend on the infectiousness of the index case, duration of the exposure, environment (crowding, poor ventilation), and virulence of the organism.

Atypical presentations and slow confirmation by culture often delay the diagnosis and treatment of patients with TB. Other reasons include an under use of tuberculin skin tests, misinterpretation of unusual chest X-rays, and waiting for culture results in patients with AFB-negative smears [6].

Chest radiography is often reliable and an important investigation. Generally, in TB, a segmental pneumonic process is seen but may be too small to visualize radiographically or complicated by atelectasis from the raised diaphragm compressing the lower lung fields in pregnancy [7, 8]. Upper zone shadowing, often bilateral and likely associated with cavitation or miliary shadowing are the classic findings. New findings like soft shadowing amongst old, fibrotic changes generally indicate a relapse. Paratracheal, mediastinal, and hilar lymphadenopathy, though not unusual in African and Indian patients with TB or in children, are generally unusual in immunocompetent adults like ours. In patients infected with HIV, the radiological appearances could often be nonspecific [9].

In our patient, the situation was complicated by a right lower lobe infiltrate. The infiltrate could have been misdiagnosed as atelectasis in a woman with a 32-week twin pregnancy. Tuberculin skin test in our patient was read as 10 mm. Five millimeters is considered to be positive in individuals with HIV, on steroid therapy, or in individuals in close contact with a person with active TB. Our patient denied any contact with TB. Larger reactions of greater than or equal to 10 mm are considered positive in recent (within the last five years) immigrants from high-prevalence countries, in individuals with diabetes, renal failure, and health-care workers, among others. Our patient falls into this category. In individuals with no known risks for TB, a positive reaction requires a 15 mm or greater induration [10].

Many patients with suspected pulmonary TB do not produce sputum spontaneously or are smear-negative for AFB. We could not obtain sputum sample in our patient due to absence of a productive cough and sputum induction was also unsuccessful. Though the BAL sample did not show any AFB, the biopsy showed caseating granuloma which could be found both in tubercular and nontubercular mycobacterial infections or fungal infections due to *Coccidioides immitis* and *Histoplasma capsulatum*. It was ultimately a DNA probe from her lung tissue that grew *M. tuberculosis* after 26 days.

With the use of PCR, nucleic acid sequences unique to *M. tuberculosis* can be detected directly in clinical specimens with better accuracy and urgency than AFB smear and culture, respectively. Probes are used for rapid identification and maximizing cost effectiveness. Used alone or in combination with other identification methods, they serve as a substitute for biochemical testing and are also more accurate [11]. Molecular tests in combination with “classic tests” can enhance the diagnostic ability particularly in pauci-bacillary infections and in patients with atypical presentations like ours.

The risk of TB to health-care workers is real. In the early 1990s, many urban US hospitals reported purified protein derivative (PPD) conversion rates in health-care workers of 3–5% [15]. A survey of US hospitals conducted by the Centers for Disease Control and Prevention (CDC) found a mean conversion rate of 1.6% [16, 17]. More recent studies have demonstrated rates of around 1% annually. Efficient control of nosocomial TB is compromised by the same difficulties complicating community control hence a high degree of suspicion is required to recognise and isolate patients with TB. An improved clinical acumen, development of rapid diagnostic tests, and the institution of early empiric therapy are desirable to control this disease. We combined the use of bronchoscopy and *M. tuberculosis* complex-PCR which provided a good diagnostic yield in our patient.

An early diagnosis of TB infection in a pregnant woman is important as better results are seen in women who are detected with TB and have been treated before the onset of pregnancy or earlier in its course, when compared with untreated patients with TB [18]. Infant and maternal mortality are between 30% and 40% in untreated active TB cases [19]. Treatment should also be initiated when the probability is moderate-to-high. Although the drugs in the initial treatment regime cross the placenta, these concentrations do not appear to have harmful effect on the fetus [20]. Pregnant women with TB should also be tested for HIV as there is a higher incidence of extrapulmonary TB and multidrug-resistant TB (MDR TB) in this set of patients [18]. Breast feeding should not be discouraged in women being treated with first-line antituberculosis drugs because the concentrations in the breast milk are subtherapeutic and too low to produce toxicity in the nursing new born. The risks of second-line medications are unknown. The effect would likely be much lower if the mother breast feeds before taking the medication. Similarly, breast milk is also inadequate as a treatment option for TB or latent TB infection in newborns [19]. Close follow up of patients is essential since current therapy for TB infection is long and suboptimal adherence may result in failure of therapy.

Congenital TB is rare with symptoms typically developing during the second or third week of life which include poor feeding, poor weight gain, cough, lethargy, and irritability. Other symptoms include fever, ear discharge, and skin lesions. Two possible routes of *M. tuberculosis* infection in utero are (a) hematogenous infection through the umbilical vein, with primary lesions in the liver and occasional porta hepatis lymphadenopathy; and (b) prenatal aspiration of infected fluid, with predominant pulmonary and gastrointestinal disease [21, 22]. The criteria for congenital TB requires the infant to have a tuberculous lesion (e.g., infiltrates on the chest radiograph or granulomas) and at least one of the following: (a) onset during the first week of life, (b) presence of a primary hepatic TB complex or caseating hepatic granulomas, (c) infection of the placenta or maternal genital tract, or (d) exclusion of postnatal transmission [22].

 The role of Bacillus of Calmette-Guérin (BCG) vaccine in preventing TB in adults is debatable due to its variable efficacy (0–80%) [23]. Its efficacy in prevention of tuberculous meningitis and miliary TB in young children has been easier to document than in adults or in the prevention of pulmonary TB in both children and adults [24]. The Centers for Disease Control has made the following recommendations concerning BCG vaccination in the US [25]. (i) BCG vaccination should be considered in infants and children 5 years if the child is exposed continually to an untreated or ineffectively treated patient with infectious pulmonary TB, if the child cannot be separated from the presence of the infectious patient or be given long-term primary preventive therapy, or if the child is exposed continually to a patient with infectious pulmonary TB caused by *M. tuberculosis* strains resistant to isoniazid and rifampin and the child cannot be separated from the presence of the infectious patient. (ii) Health-care workers in high-risk settings should be considered on an individual basis in settings in which a high percentage of TB patients are infected with *M. tuberculosis* strains resistant to both isoniazid and rifampin, transmission and subsequent infection are likely and where comprehensive TB infection-control precautions have been implemented and have not been successful.

## 4. CONCLUSION

Our case emphasizes the significance of identifying infectious diseases like TB in developing countries due to a change in demographics. It also underscores the importance of an early diagnosis and treatment even in the backdrop of confounding evidence in pregnant women due to better maternal and perinatal outcomes. A heightened index of suspicion and awareness of the rapid advances and innovations made in the diagnosis of TB is also essential for better control of this disease.

## Figures and Tables

**Figure 2:**
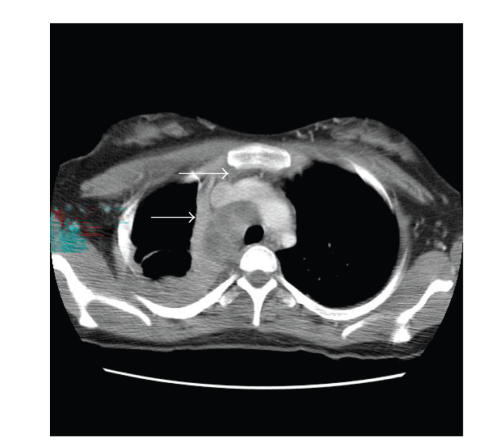
Computer tomography of the chest. Extensive mediastinal soft tissue mass in the right paratracheal region extending into the hilar and subcarinal region.

**Table 1:** Accuracy of various methods of diagnosis of tuberculosis with their sensitivities and specificities [12–14].

Testing methods	Sensitivity	Specificity
(1) Chest X-ray	66–77	66–76
(2) TST	75–90	75–90
(3) RD1-based Gammainterferon tests	80–95	95–100
(4) Acid fast smear	60	92
(5) Culture	90	99.6
(6) PCR (smear-positive sample)	96	85
(7) (Smear-negative sample)	66	98
